# The Impact of Initial COVID-19 Episode Inflammation Among Adults on Mortality Within 12 Months Post-hospital Discharge

**DOI:** 10.3389/fmed.2022.891375

**Published:** 2022-05-12

**Authors:** Arch G. Mainous, Benjamin J. Rooks, Frank A. Orlando

**Affiliations:** ^1^Department of Community Health and Family Medicine, University of Florida, Gainesville, FL, United States; ^2^Department of Health Services Research Management, and Policy, University of Florida, Gainesville, FL, United States

**Keywords:** COVID-19, cohort, mortality, post-acute COVID-19, inflammation

## Abstract

**Background:**

Inflammation in the initial COVID-19 episode may be associated with post-recovery mortality. The goal of this study was to determine the relationship between systemic inflammation in COVID-19 hospitalized adults and mortality after recovery from COVID-19.

**Methods:**

An analysis of electronic health records (EHR) for patients from 1 January, 2020 through 31 December, 2021 was performed for a cohort of COVID-19 positive hospitalized adult patients. 1,207 patients were followed for 12 months post COVID-19 episode at one health system. 12-month risk of mortality associated with inflammation, C-reactive protein (CRP), was assessed in Cox regressions adjusted for age, sex, race and comorbidities. Analyses evaluated whether steroids prescribed upon discharge were associated with later mortality.

**Results:**

Elevated CRP was associated other indicators of severity of the COVID-19 hospitalization including, supplemental oxygen and intravenous dexamethasone. Elevated CRP was associated with an increased mortality risk after recovery from COVID-19. This effect was present for both unadjusted (HR = 1.60; 95% CI 1.18, 2.17) and adjusted analyses (HR = 1.61; 95% CI 1.19, 2.20) when CRP was split into high and low groups at the median. Oral steroid prescriptions at discharge were found to be associated with a lower risk of death post-discharge (adjusted HR = 0.49; 95% CI 0.33, 0.74).

**Discussion:**

Hyperinflammation present with severe COVID-19 is associated with an increased mortality risk after hospital discharge. Although suggestive, treatment with anti-inflammatory medications like steroids upon hospital discharge is associated with a decreased post-acute COVID-19 mortality risk.

## Introduction

The impact of coronavirus disease 2019 (COVID-19) has been immense. In terms of directly measured outcomes, as of February, 2022, worldwide more than 5.9 million people have died from directly linked COVID-19 episodes. More than 950,000 direct deaths from COVID-19 have been documented in the United States (1). Some evidence has suggested that some patients with COVID-19 may be at risk for developing health problems after the patient has recovered from the initial episode (2–4). Common sequelae that have been noted are fatigue, shortness-of-breath, and brain fog. Perhaps more concerningly, in addition to these symptoms, several studies have shown that following recovery from the initial COVID-19 episode, some patients are at risk for severe morbidity and mortality (5–8). Patients who have recovered from COVID-19 are at increased risk for hospitalization and death within 6–12 months after the initial episode. This morbidity and mortality is typically not listed or considered as a COVID-19 linked hospitalization or death in the medical records and thus are underreported as a post-acute COVID-19 sequelae.

The reason for this phenomenon of severe outcomes as post-acute sequelae of COVID-19 is not well understood. Early in COVID-19 episode, the disease is primarily driven by the replication of SARS-CoV-2. COVID-19 also exhibits a dysregulated immune/inflammatory response to SARS-CoV-2 that leads to tissue damage. The downstream impact of the initial COVID-19 episode is consistently higher in people with more severe acute infection (5, 6, 9). Cytokine storm, hyperinflammation, and multi-organ failure have also been indicated in patients with a severe COVID-19 episode (10). Cerebrospinal fluid samples indicate neuroinflammation during acute COVID-19 episodes (11). Moreover, even 40–60 days post-acute COVID-19 infection there is evidence of a significant remaining inflammatory response in patients (12). Thus, it could be hypothesized that the hyperinflammation that some COVID-19 patients have during the initial COVID-19 episode creates a systemic damage to multiple organ systems (13, 14). Consequently, that hyperinflammation and the corresponding systemic damage to multiple organ systems may lead to severe post-acute COVID-19 sequelae.

Following from this hyperinflammation, the use of steroids as anti-inflammatory treatments among patients with high inflammation during the initial COVID-19 episode may do more than just help in the initial episode but may act as a buffer to the downstream morbidity and mortality from the initial COVID-19 episode (14, 15).

The purpose of this study was to examine the relationship between substantial systemic inflammation, as measured by C-reactive protein (CRP), with post-acute COVID-19 sequelae among patients hospitalized with COVID-19. This 12-month mortality risk was examined in a longitudinal cohort of patients who tested positive for COVID-19 as determined by Polymerase Chain Reaction (PCR) testing within a large healthcare system.

## Methods

The data for this project comes from a de-identified research databank containing electronic health records (EHR) of patients tested for or diagnosed with COVID-19 in any setting in the University of Florida (UF) Health system. Usage of the databank for research is not considered human subjects research, and IRB review was not required to conduct this study.

### Definition of Cohort

The cohort for this study consisted of all adult patients aged 18 and older who were tested for COVID-19 between January 01, 2020 and December 31, 2021 within the UF Health system, in any encounter type (ambulatory, Emergency Department, inpatient, etc.). Although a patient in the cohort could have had a positive test administered in any of these settings, a patient was only included into the cohort if that patient experienced a hospitalization for COVID-19. Since this study included data from the early stages of the pandemic before consistent coding standards for documenting COVID-19 in the EHR had been established, a patient was considered to have been hospitalized for COVID-19 if they experienced any hospitalization within 30 days of a positive test for COVID-19. The databank contained EHR data for all patients in the cohort current through December 31, 2021. COVID-19 diagnosis was validated by PCR. Baseline dates for COVID-19 positive patients were established at the date of their earliest recorded PCR-confirmed positive COVID-19 test. Each patient was only included once in the analysis. For patients with multiple COVID-19 tests, if at least one test gave a positive result, the patient was classified as COVID-19 positive, and the date of their earliest positive COVID-19 test result was used as their baseline date. Patients who did not have a positive COVID-19 test were not included in the analysis. Patients were tested in the context of seeking care for COVID-19; the tests were not part of general screening and surveillance.

Only patients with at least 365 days of follow-up time after their baseline date were retained in the cohort. Patients with more than 365 days of follow-up were censored at 365 days. The cohort was also left censored at the 30-day mark post-hospital discharge to ensure that health care utilization was post-acute and not part of the initial COVID-19 episode of care (e.g., not a readmission).

### Inflammation

C-reactive protein (CRP) was used as the measure of inflammation in this study. The UF Health laboratory measured CRP in serum using latex immunoturbidimetry assay. CRP measures were sourced from patient EHR data. The cohort was restricted to only include patients with at least one CRP measurement within their initial COVID-19 episode of care (between the date of their initial positive COVID-19 test and the left-hand censoring date). For patients with multiple measurements of CRP, the maximum value available was used.

### Steroids

Intravenous dexamethasone during their initial COVID-19 hospitalization was assessed. Prescriptions for oral steroids (tablets of dexamethasone) that were prescribed either at or post-hospital discharge for their initial COVID-19 episode of care were included into the analysis. Prescriptions were identified using RxNorm codes available in each patient's EHR.

### Severity of Initial COVID-19 Hospitalization

We also measured the severity of the initial episode of COVID-19 hospitalization. This severity should track with the level of inflammation in the initial COVID-19 episode. We used the National Institutes of Health's “Therapeutic Management of Hospitalized Adults With COVID-19” disease severity levels and definitions (16). The recommendations are based on four ascending levels: hospitalized but does not require supplemental oxygen, hospitalized and requires supplemental oxygen, hospitalized and requires supplemental oxygen through a high-flow device or noninvasive ventilation, hospitalized and requires mechanical ventilation or extracorporeal membrane oxygenation. For this study, because of the general conceptual model of severity moving from no supplemental oxygen to supplemental oxygen to mechanical ventilation, we collapsed the two supplemental non-mechanical ventilation oxygen into one intermediate category of severity.

### Outcome Variables

The primary outcome investigated in this study was the 365-day all-cause mortality. Mortality data was sourced both from EHR data and the Social Security Death Index (SSDI), allowing for the assessment of deaths which occurred outside of UF's healthcare system. When conflicting dates of death were observed between the EHR and SSDI, the date recorded in the patient's medical record was used. Patients who died within their 365-day follow-up window were censored at the date of their recorded death. The cause of death was not available in the EHR based database and was not routinely and reliably reported in either the SSDI or EHR. We were unable to estimate the cause of death.

### Comorbidities

Comorbidities and demographic variables which could potentially confound the association between inflammation represented by CRP and mortality post-acute COVID-19 were collected at baseline for each member of the cohort. Demographic variables included patient age, race, ethnicity, and sex. The Charlson Comorbidity Index was also calculated, accounting for the conditions present for each patient at their baseline. The Charlson Comorbidity Index was designed to be used to predict 1-year mortality and is a widely used measure to account for comorbidities (17).

### Analysis

CRP was evaluated using descriptive statistics. We performed a median split of the CRP levels and defined elevated inflammation as a CRP level at or above the median and levels below the median as low inflammation. Additionally, as a way to examine greater separation between high and low inflammation, we segmented CRP levels into tertiles and categorized elevated inflammation as the top tertile and compared it to the first tertile by chi-square tests.

CRP level was also cross classified by severity of COVID-19 hospitalization and associations between the two variables were assessed using one-way ANOVA tests.

Kaplan-Meier curves comparing the survival probabilities of the high and low inflammation groups were created and compared using a log-rank test. Hazard ratios for the risk of death for post-acute COVID-19 complications by COVID-19 status were determined using Cox proportional hazard models. We obtained hazard ratios for mortality based on tertile and median splits of CRP. These analyses were then modified to control for age, sex, race, ethnicity, and the Charlson Comorbidity Index.

Additional analyses stratified by use of steroids were performed to compare the strength of the association between inflammation and death. The proportional hazards assumption was confirmed by inspection of the Schoenfeld residual plots for each variable included in the models and testing of the time-dependent beta coefficients. Analyses were conducted using the survival package in R v4.0.5.

## Results

A total of 1,207 patients were included in the final cohort (Table 1). The characteristics of the patients are featured in Table 1. The mean CRP rises with the severity of illness in these COVID-19 inpatients. The mean CRP in the lowest severity (no supplemental oxygen) is 59.4 mg/L (SD = 61.8 mg/L), while the mean CRP in the intermediate severity group (supplemental oxygen) is 126.9 mg/L (SD = 98.6 mg/L), and the mean CRP in the highest severity group (ventilator or ECMO) is 201.2 mg/L (SD = 117.0 mg/L) (*p* < 0.001). Similarly, since dexamethasone is only recommended for the most severe patients with COVID-19, patients with dexamethasone had higher CRP (158.8 mg/L; SD = 114.9 mg/L) than those not on Dexamethasone (102.8 mg/L; SD = 90/8 mg/L) (*p* < 0.001).

**Table 1:** Characteristics of the patients in the cohort.

	**Total (*n* = 1,207)**	**Top tertile for CRP (*n* = 411)**	**Bottom tertile for CRP (*n* = 398)**	***p*** -value^*^
**No. (%) with data**				
All-cause deaths	175 (14.5%)	74 (18.0%)	50 (12.6%)	0.040
Steroid prescriptions on discharge	509 (42.2%)	167 (40.6%)	144 (36.2%)	0.219
**Supplemental oxygen**				
No oxygen	254 (21.0%)	30 (7.3%)	160 (40.2%)	<0.001
Supplemental oxygen	806 (66.8%)	286 (69.6%)	226 (56.8%)	<0.001
Ventilator	143 (11.8%)	94 (22.9%)	9 (2.3%)	<0.001
Dexamethasone in the COVID-19 hospitalization	412 (34.1%)	192 (46.7%)	76 (19.1%)	<0.001
Male	538 (44.6%)	191 (46.5%)	165 (41.5%)	0.172
Non-Hispanic White	490 (40.6%)	139 (33.8%)	187 (47.0%)	<0.001
Non-Hispanic Black	599 (49.6%)	229 (55.7%)	182 (45.7%)	0.006
**Age**				
Under 65	726 (60.1%)	237 (57.7%)	251 (63.1%)	
65+	481 (39.9%)	174 (42.3%)	147 (36.9%)	0.134
**Charlson comorbidity index score**				
0–1	526 (43.6%)	169 (41.1%)	176 (44.2%)	0.412
2–3	293 (24.3%)	98 (23.8%)	95 (23.9%)	0.999
4+	388 (32.1%)	144 (35.0%)	127 (31.9%)	0.389

* *Results from Chi-squared tests*.

**Figure 1:**
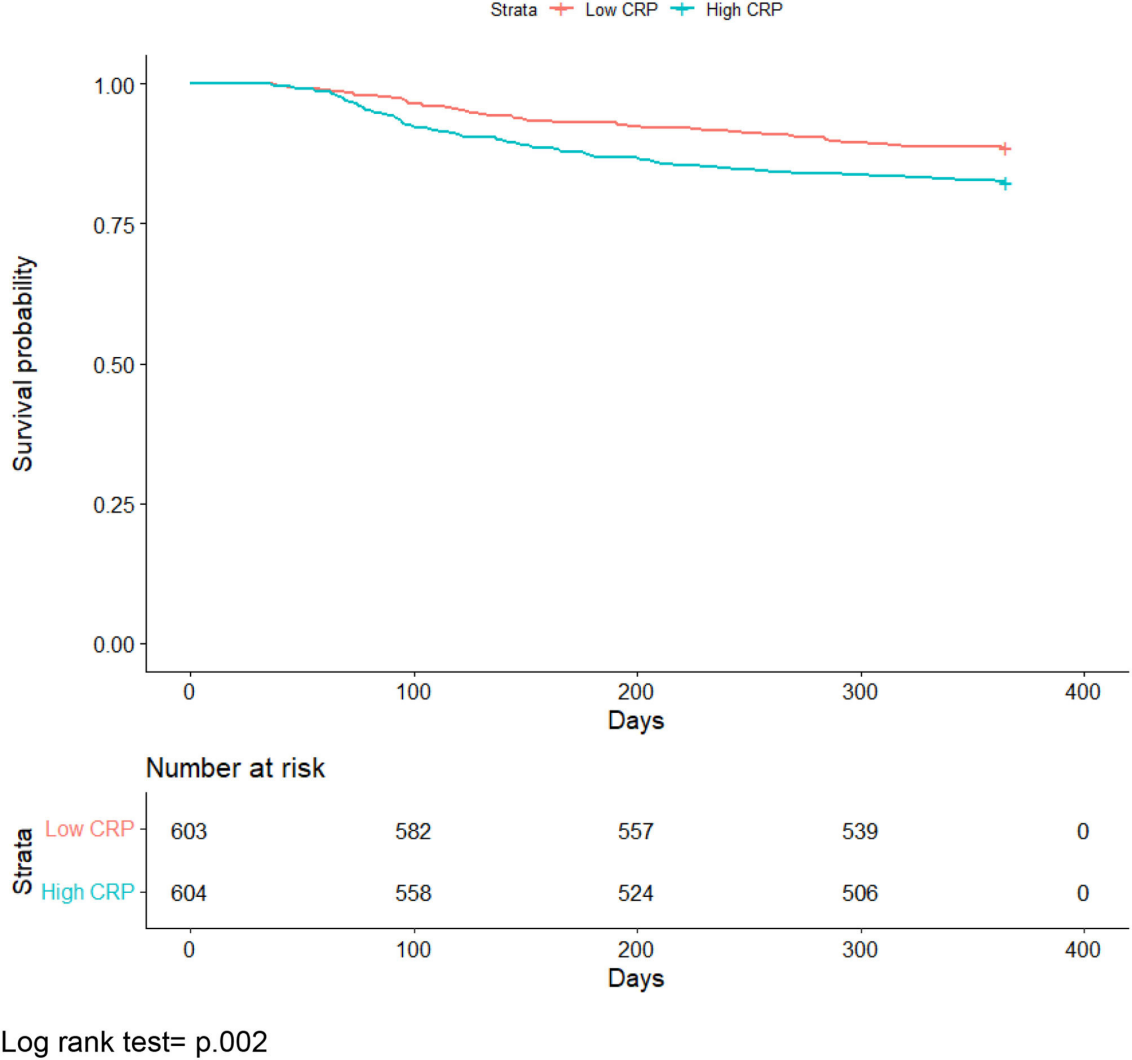
All-cause mortality Kaplan-Meier curve comparing individuals with median or greater vs. below median C-reactive protein levels. Log rank test = p.002.

Figure 1 presents the Kaplan-Meier curves comparing the risk of mortality by inflammation over time. A log-rank test indicated there was a statistically significant difference in survival probabilities between the two groups (*p* = 0.002).

Table 2 shows the relationship between levels of inflammation and mortality post-recovery from COVID-19. In both unadjusted and adjusted analyses, elevated inflammation has a significantly increased risk compared to those with low inflammation in the initial COVID-19 episode. This finding of higher inflammation during the initial COVID-19 hospitalization and increased mortality risk after recovery was similar when CRP was split at the median and when the third tertile of CRP was compared to the first tertile of CRP. The proportional hazards assumption was met when the Schoenfeld plots.

**Table 2:** All-cause mortality hazard ratios by inflammation and steroid use.

	**Hazard ratios (95% CI)**	
	**Unadjusted**	**Adjusted^**a**^**
**Inflammation**		
Top 50% vs. bottom 50% for CRP	1.60 (1.18, 2.17)	1.61 (1.19, 2.20)
Top tertile vs. bottom tertile for CRP	1.50 (1.05, 2.15)	1.61 (1.12, 2.32)
**Steroid use**		
Steroid prescriptions at discharge	0.43 (0.29, 0.63)	0.49 (0.33, 0.74)

a *Models were adjusted for age, race/ethnicity, sex, and the Charlson Comorbidity Index*.

We examined the hypothesized relationship that potentially decreasing inflammation in COVID-19 patients with an initial severe episode may have beneficial downstream effects on post-acute COVID-19 sequelae. Oral steroid prescriptions at discharge among these hospitalized COVID-19 patients were found to be associated with a lower risk of death post-discharge (Table 2).

## Discussion

The results of this study reaffirm the importance of post-acute COVID-19 sequelae. This study is the first to show the impact of inflammation in the initial COVID-19 hospitalization episode on downstream mortality after the patient has recovered. This expands our understanding of post-acute COVID-19 sequelae by providing a better concept of why certain patients have post-acute COVID-19 mortality risk.

Previous studies have shown that patients who are hospitalized with COVID-19 have an increased risk of mortality 12 months after recovery (5). Those findings suggest that prevention of COVID-19 hospitalizations is of paramount importance. However, some patients will be hospitalized. The finding that elevated inflammation during the initial hospitalization episode is associated with mortality risk after recovery suggests that it may be worthwhile treating the viral episode but also consider treating the hyperinflammation. The NIH recommendations for care of COVID-19 hospitalized patients recommend steroids only for patients who need supplemental oxygen (16). The finding that the use of steroids prescribed upon discharge from the hospital and the corresponding reduced risk of mortality indicate that treating inflammation after the acute COVID-19 episode may act as a buffer to the downstream mortality risk from the initial COVID-19 episode (14, 15). Perhaps this requires a reconceptualization of COVID-19 as both an acute disease and potentially a chronic disease because of the lingering risks. Future research is needed to see if ongoing treatment for inflammation in a clinical trial has positive benefits.

There are several strengths and limitations to this study. The strengths of this study include the PCR validated COVID-19 tests at baseline for the cohort. Further, the linked electronic health record allows us to look not only at health care utilization like hospitalizations and both inpatient and outpatient medication but also laboratory tests like CRP levels. The cohort also allows us to have a substantial follow-up time.

In terms of limitations, the first that needs to be considered is that the analysis was based on hospitalized patients seen in one health system with a regional catchment area. Although more than 1200 hospitalized patients with PCR validated COVID-19 diagnoses were included in the analysis, and the cohort was followed for 12 months, the primary independent variable was systemic inflammation which should not be substantially affected by region of the country. Second, the data are observational. Thus, the analyses related to steroids and downstream mortality require a clinical trial to confirm these suggestive findings. Third, we did not have death certificates available to us to compute cause of death. The Social Security Death Index in partnership with the EHR allows us to be confident that the patient died and so we have a strong measure of all-cause mortality but we were unable to determine specific causes of death within this database. Fourth, although there are a variety of other markers of inflammation (e.g., D dimer, IL 6), CRP is one of the most robust measures of systemic inflammation. Moreover, it is much more widely used and was the most prevalent marker among the patients in the study.

In conclusion, hyperinflammation present with severe COVID-19 is associated with an increased mortality risk after hospital discharge. Although suggestive, treatment with anti-inflammatory medications like steroids upon hospital discharge is associated with a decreased post-acute COVID-19 mortality risk. This suggests that treating inflammation may also benefit other post-acute sequelae like long COVID. A reconceptualization of COVID-19 as both an acute and chronic condition may be useful.

## Data Availability Statement

The original contributions presented in the study are included in the article/supplementary material, further inquiries can be directed to the corresponding author/s.

## Author Contributions

AM, BR, and FO designed the study, generated hypotheses, and interpreted the data. BR analyzed the data with input from AM and FO. AM drafted the initial manuscript and he is the guarantor. All authors had full access to all the data in the study and had final responsibility for the decision to submit for publication, contributed to the article, and approved the submitted version.

## Funding

The research reported in this publication was supported by the National Center for Advancing Translational Sciences of the National Institutes of Health under University of Florida Clinical and Translational Science Awards UL1TR000064 and UL1TR001427.

## Conflict of Interest

The authors declare that the research was conducted in the absence of any commercial or financial relationships that could be construed as a potential conflict of interest.

## Publisher's Note

All claims expressed in this article are solely those of the authors and do not necessarily represent those of their affiliated organizations, or those of the publisher, the editors and the reviewers. Any product that may be evaluated in this article, or claim that may be made by its manufacturer, is not guaranteed or endorsed by the publisher.
